# Supplementary material for: Trophodynamics of the Antarctic toothfish (Dissostichus mawsoni) in the Antarctic Peninsula: Ontogenetic changes in diet composition and prey fatty acid profiles
Source: PLoS One. 2023 Oct 5;18(10):e0287376. doi: 10.1371/journal.pone.0287376 (PMC10553334; doi:10.1371/journal.pone.0287376)
Supplement: S1 Table — (DOCX) [file pone.0287376.s001.docx]

**S1 Table**. Date, haul number, geographic position and depth (m) of fishing hauls carried out by the Ukrainian commercial vessel Calipso in the Antarctic Peninsula (Subarea 48.1) during 2019/20 and 2020/21.

| **Date** | **Haul number** | **Geographic position** | **Research block** | **Depth (m)** |
| --- | --- | --- | --- | --- |
| 02.02.2020 | 1 | 63°56,11’S - 53°51,14’W | 48.1_2 | 924 |
| 03.02.2020 | 2 | 63°56,90’S - 53°41,20’W | 48.1_2 | 1183 |
| 04.02.2020 | 3 | 62°29,90’S - 53°29,80’W | 48.1_1 | 1268 |
| 04.02.2020 | 4 | 62°36,20’S - 53°27,40’W | 48.1_1 | 1040 |
| 04.02.2020 | 5 | 62°29,90’S - 53°47,30’W | 48.1_1 | 1227 |
| 06.02.2020 | 6 | 62°26,00’S - 53°33,20’W | 48.1_1 | 1560 |
| 06.02.2020 | 7 | 62°04,95’S - 53°35,45’W | 48.1_1 | 1100 |
| 06.02.2020 | 8 | 62°10,32’S - 53°31,14’W | 48.1_1 | 1400 |
| 07.02.2020 | 9 | 62°19,46’S - 53°38,02’W | 48.1_1 | 1020 |
| 18.02.2021 | 1 | 63°58,22’S - 53°44,71’W | 48.1_2 | 976 |
| 18.02.2021 | 2 | 63°53,45’S - 52°22,44’W | 48.1_2 | 1260 |
| 19.02.2021 | 3 | 63°57,61’S - 53°43,00’W | 48.1_2 | 1250 |
| 19.02.2021 | 4 | 63°59,59’S - 53°19,30’W | 48.1_2 | 1170 |
| 20.02.2021 | 5 | 63°57,99’S -52°50,42’W | 48.1_2 | 1550 |
| 20.02.2021 | 6 | 63°52,02’S - 52°53,18’W | 48.1_2 | 1270 |
| 22.02.2021 | 8 | 63°11,96’S - 51°27,52’W | 48.1_2 | 1266 |
| 23.02.2021 | 9 | 63°12,94’S - 50°51,62’W | 48.1_2 | 1235 |
| 22.02.2021 | 10 | 63°17,81’S - 50°31,18’W | 48.1_2 | 1180 |
| 23.02.2021 | 11 | 63°21,11’S - 50°52,14’W | 48.1_2 | 1371 |
| 25.02.2021 | 14 | 63°01,45’S - 52°06,41’W | 48.1_2 | 1075 |
| 25.02.2021 | 15 | 62°52,16’S - 52°17,05’W | 48.1_1 | 1093 |
