# Supplementary material for: Trophodynamics of the Antarctic toothfish (Dissostichus mawsoni) in the Antarctic Peninsula: Ontogenetic changes in diet composition and prey fatty acid profiles
Source: PLoS One. 2023 Oct 5;18(10):e0287376. doi: 10.1371/journal.pone.0287376 (PMC10553334; doi:10.1371/journal.pone.0287376)
Supplement: S2 Table — Degrees of freedom (d.f.), Log-likelihood (LL), Akaike Information Criteria (AIC) and increments of each model in comparison with the best model (ΔAIC) are shown. (DOCX) [file pone.0287376.s002.docx]

**S2 Table.** Factors tested in MGLM to best explain the amount of variation in prey specific abundance in the stomach content of the Antarctic toothfish in the Antarctic Peninsula. Degrees of freedom (d.f.), Log-likelihood (LL), Akaike Information Criteria (AIC) and increments of each model in comparison with the best model (ΔAIC) are shown.

| **Factors** | **d.f.** | **LL** | **AIC** | **ΔAIC** |
| --- | --- | --- | --- | --- |
| Size-class | 129 | -936.20 | 990.20 | 30.28 |
| Fishing season | 130 | -951.62 | 987.62 | 27.70 |
| Sex | 130 | -987.45 | 1023.46 | 63.53 |
| Size-Class x Fishing Season x Sex | 120 | -811.24 | 1027.24 | 67.32 |
| Size-Class + Fishing Season + Sex | 127 | -874.46 | 964.46 | 4.54 |
| Size-Class x Fishing Season | 126 | -864.75 | 972.75 | 12.83 |
| Size-Class + Fishing Season | 128 | -887.92 | 959.92 | 0.00 |
