# Supplementary material for: Trophodynamics of the Antarctic toothfish (Dissostichus mawsoni) in the Antarctic Peninsula: Ontogenetic changes in diet composition and prey fatty acid profiles
Source: PLoS One. 2023 Oct 5;18(10):e0287376. doi: 10.1371/journal.pone.0287376 (PMC10553334; doi:10.1371/journal.pone.0287376)
Supplement: S3 Table — (DOCX) [file pone.0287376.s003.docx]

**S3 Table.** Post hoc pairwise comparisons of manyglm analyses, between size-class categories.

| **Post hoc pairwise comparisons** | **Sum of LR statistic** | **p-value** |
| --- | --- | --- |
| G2 vs G3 | 31.462 | 0.004 |
| G1 vs G3 | 17.231 | 0.061 |
| G1 vs G2 | 8.254 | 0.414 |
