# Supplementary material for: Trophodynamics of the Antarctic toothfish (Dissostichus mawsoni) in the Antarctic Peninsula: Ontogenetic changes in diet composition and prey fatty acid profiles
Source: PLoS One. 2023 Oct 5;18(10):e0287376. doi: 10.1371/journal.pone.0287376 (PMC10553334; doi:10.1371/journal.pone.0287376)
Supplement: S4 Table — The average dissimilarity (Av. diss, %) between groups is shown in the first row. FAs are ordered according to their percentage contribution (Contrib%) of the total dissimilarity with a 50% cutoff level. (DOCX) [file pone.0287376.s004.docx]

**S4 Table**. SIMPER results of fatty acid profile %FAs data with pairwise tests between the four identified prey groups. The average dissimilarity (Av. diss, %) between groups is shown in the first row. FAs are ordered according to their percentage contribution (Contrib%) of the total dissimilarity with a 50% cutoff level.

|  | **Fatty acid** | **Contrib%** | **Cumulative %** |
| --- | --- | --- | --- |
| ***Macrouridae - Anotopteridae*** | C20:4n6 ARA | 11.64 | 11.64 |
| ***Av. diss = 10.47*** | C18:4n3 | 11.40 | 23.04 |
|  | C18:1n7 | 10.31 | 33.35 |
|  | C22:6n3 DHA | 9.24 | 42.59 |
|  | C18:1 n9cis | 8.27 | 50.86 |
| ***Macrouridae - Channichthyidae*** | C22:6n3 DHA | 9.52 | 9.52 |
| ***Av. diss = 16.51*** | C16:1n7 | 7.97 | 17.49 |
|  | C18:1 n9cis | 7.89 | 25.38 |
|  | C14:0 | 7.81 | 33.19 |
|  | C18_4n3 | 7.30 | 40.49 |
|  | C22:1n9 | 6.66 | 47.15 |
|  | C20:1n9 | 5.38 | 52.54 |
| ***Anotopteridae - Channichthyidae*** | C22:6n3 DHA | 9.69 | 9.69 |
| ***Av. diss = 15.67*** | C16:1n7 | 8.45 | 18.15 |
|  | C14:0 | 8.41 | 26.56 |
|  | C18:1 n9cis | 8.05 | 34.61 |
|  | C18:4n3 | 7.58 | 42.19 |
|  | C22:1n9 | 6.87 | 49.06 |
|  | C20:4n6 ARA | 6.31 | 55.37 |
| ***Macrouridae - Chephalopoda*** | C18:1 n9cis | 13.4 | 13.40 |
| ***Av. diss = 17.59*** | C20:1 n9 | 9.38 | 22.78 |
|  | C22:6n3 DHA | 8.28 | 31.05 |
|  | C18:2 n6cis | 8.24 | 39.29 |
|  | C16:1n7 | 7.37 | 46.66 |
|  | C18:4n3 | 5.87 | 52.53 |
| ***Anotopteridae - Cephalopoda*** | C20:1n9 | 13.17 | 13.17 |
| ***Av. diss = 17.67*** | C20:4n6 ARA | 11.67 | 24.85 |
|  | C18:1 n9cis | 10.49 | 35.33 |
|  | C18:2 n6cis | 8.33 | 43.66 |
|  | C18:1n7 | 7.60 | 51.27 |
| ***Channichthydae - Cephalopoda*** | C20:1 n9 | 10.84 | 10.84 |
| ***Av. diss = 23.21*** | C18:1 n9cis | 8.64 | 19.48 |
|  | C18:2 n6cis | 8.04 | 27.52 |
|  | C22:6n3 DHA | 8.02 | 35.54 |
|  | C16:1n7 | 7.37 | 42.92 |
|  | C14:0 | 5.75 | 48.67 |
|  | C18:4n3 | 5.18 | 53.85 |
