# Supplementary material for: Trophodynamics of the Antarctic toothfish (Dissostichus mawsoni) in the Antarctic Peninsula: Ontogenetic changes in diet composition and prey fatty acid profiles
Source: PLoS One. 2023 Oct 5;18(10):e0287376. doi: 10.1371/journal.pone.0287376 (PMC10553334; doi:10.1371/journal.pone.0287376)
Supplement: S1 Fig — (DOCX) [file pone.0287376.s005.docx]

**
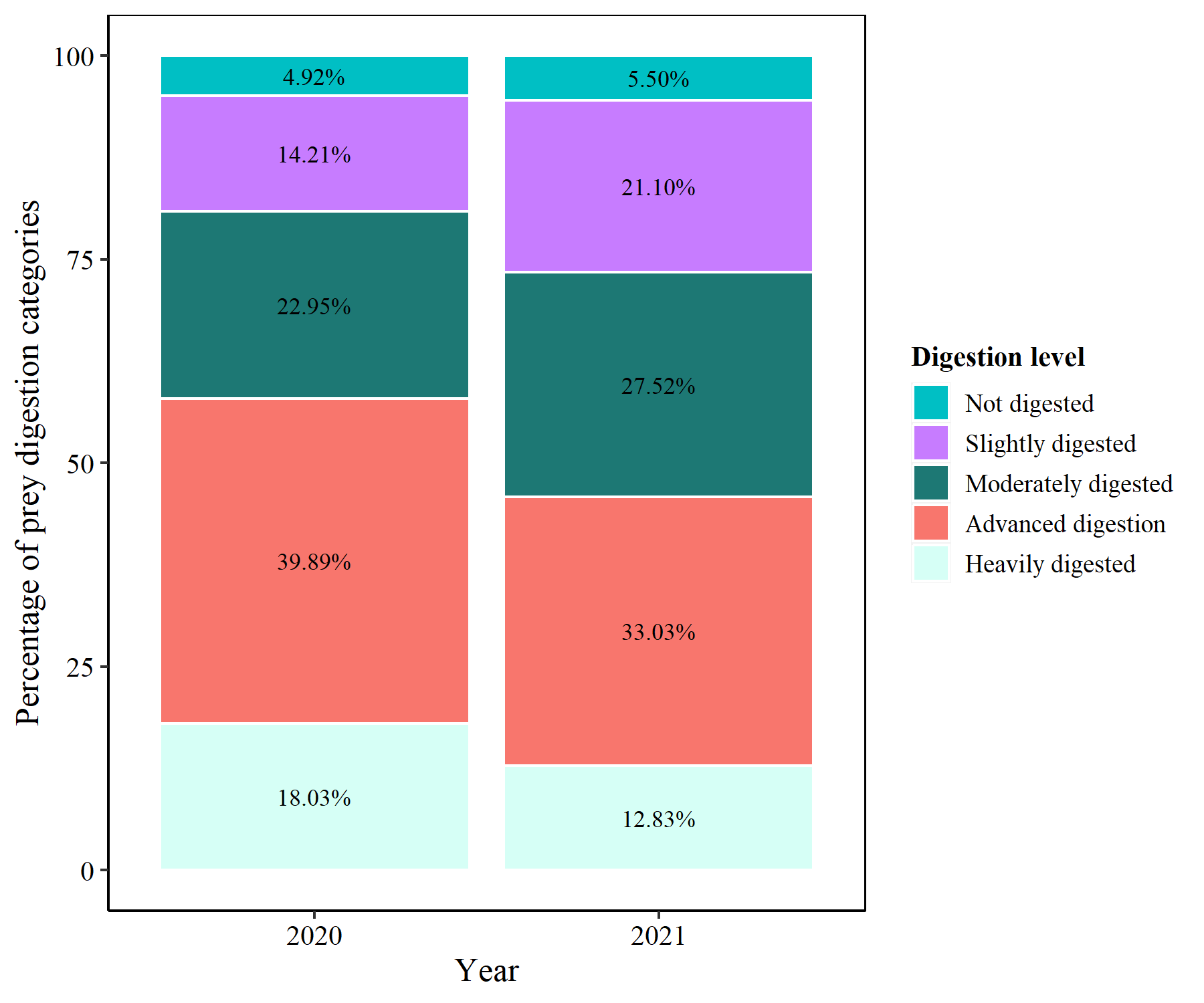
**

**S1 Figure**. Digestion level of prey items in the stomach of *Dissostichus mawsoni* collected in the northern tip of the Antarctic Peninsula during fishing season 2019/20 (n=186) and 2020/21 (n =140).
