# Supplementary material for: Trophodynamics of the Antarctic toothfish (Dissostichus mawsoni) in the Antarctic Peninsula: Ontogenetic changes in diet composition and prey fatty acid profiles
Source: PLoS One. 2023 Oct 5;18(10):e0287376. doi: 10.1371/journal.pone.0287376 (PMC10553334; doi:10.1371/journal.pone.0287376)
Supplement: Attachment — Submitted filename: renamed_c1ecc.docx [file pone.0287376.s007.docx]

**Trophodynamics of the Antarctic toothfish (*Dissostichus mawsoni*) in the Antarctic Peninsula: Ontogenetic changes in diet composition and prey fatty acid profiles**

**Karina Pérez-Pezoa^1^, César A. Cárdenas^2,3^, Marcelo González-Aravena^2^, Pablo Gallardo^4^, Alí Rivero^4^, Vicente Arriagada^5^, Kostiantyn Demianenko^6^, Pavlo Zabroda^7^, Francisco Santa Cruz^2*^**

1 Departamento de Ecología, Pontificia Universidad Católica de Chile, Santiago, Chile.

2 Departamento Científico, Instituto Antártico Chileno, Plaza Muñoz Gamero, 1055, Punta Arenas, Chile.

3 Millennium Institute Biodiversity of Antarctic and Subantarctic Ecosystems (BASE), Santiago, Chile.

4 Departamento de Ciencias Agropecuarias y Acuícolas, Universidad de Magallanes, Punta Arenas, Chile.

5 Departamento de Microbiología, Universidad de Concepción, Concepción, Chile.

6 Institute of Fisheries and Marine Ecology (IFME), 71118 Berdyansk, Ukraine.

*corresponding author: fsantacruz[@inach.cl](mailto:lkruger@inach.cl)

**Abstract**

Antarctic toothfish (*Dissostichus mawsoni*) is the largest notothenioid species in the Southern Ocean, playing a keystone role in the trophic web as a food source for marine mammals and a top predator in deep-sea ecosystems. Most ecological knowledge on this species relies on samples from areas where direct fishing is allowed, whereas in areas closed to fishing, such as the Antarctic Peninsula (AP), there are still key ecological gaps to ensure effective conservation, especially regarding our understanding of its trophic relationships within the ecosystem. Here, we present the first comprehensive study of the feeding behavior of Antarctic toothfish caught in the northern tip of the AP, during two consecutive fishing seasons (2020-2021). Stomach content was analyzed according to size-classes, sex, and season. Macroscopic morphological analysis was used to identify prey, whereas DNA analysis was used in highly digested prey items. Fatty acid analysis was conducted to determine the prey's nutritional composition. The diet mainly consisted of Macrouridae, Cephalopoda, Anotopteridae, and Channichthyidae. Other rare prey found were crustaceans, and penguin remains. While sex had no effect on diet, size-class and fishing season influenced the prey composition. From 27 fatty acid profiles identified, we observed two different prey groups of fishes (integrated by families Anotopteridae, Macrouridae, and Channichthyidae) and cephalopods. Our results revealed a narrow range of prey items typical of a generalist predator that probably consumes the most abundant prey. Understanding the diet and trophic relationships of Antarctic toothfish is critical for a better comprehension of its role in the benthic-demersal ecosystem of the AP, key for ecosystemic fisheries management and relevant to understand and predict the effect of climate change on this species.

**Keywords:** Trophic ecology, prey identification, notothenioid, top predator, Southern Ocean, ecosystem-based management

**Introduction**

The Southern Ocean is characterized by extreme physical conditions that have shaped a unique, endemic, and highly adapted fauna [1,2]. A good example is its fish fauna, characterized by relatively low species richness and diversity, highly dominated by the suborder Notothenioidei, a group highly adapted to cold waters [1,3]. Among this group, *Dissostichus mawsoni,* commonly known as Antarctic toothfish (hereafter TOA), is a species circumpolar distributed above 60°S latitude, at water depths up to 3000 meters along the shelf and continental slope, with temperatures below 0°C [4–6]. Individuals can exceed 2 meters length and 100 kg weight [7,8], lasting over 30 years with a first sexual maturation between 12 and 16 years of age [5,9]. By far is the larger fish species, playing a key ecological role in the trophic web, both as a food source for marine mammals such as cetaceans [10] and seals [11], and as the top predator in deep-sea ecosystems, structuring the size and population dynamics of its prey species through a trophic cascade [8,12].

TOA is a valuable fishing resource targeted by an international bottom-set longline fleet that is managed by the Commission for the Conservation of Antarctic Marine Living Resources (CCAMLR), whose main objective is to achieve a balance between the rational use and conservation of fishing stocks. CCAMLR also promotes ecosystem-based fisheries management, through conservation measures based on exploitation levels that ensure recruitment stability and ecological relationships functioning to avoid irreversible changes in the marine ecosystems [13]. At present, TOA is targeted throughout almost their entire distribution range, with a total annual catch of around 4000 tons occurring mostly in the Ross Sea, East Antarctica, and the Weddell Sea [14]. The only exception is the Antarctic Peninsula (AP, FAO Statistical Subarea 48.1), where direct fishing for notothenioid is prohibited, due to population collapses after overexploitation in the 1970s and 1980s and still no evidence of population recovery [15]. Nowadays, CCAMLR only allows exploratory fisheries with minimum catch limits aimed to obtain information on the biological and ecological interspecific information for ecosystem-based purposes [8,16–18].

The prohibition of regular fishing activities has determined gaps of knowledge regarding the population and community dynamics of the TOA in Subarea 48.1; thus CCAMLR Scientific Committee has asked for new studies to reduce uncertainty [19]. Information on trophic dynamics is scarce, and diet analysis based on prey composition is necessary to understand population dynamics and its interaction with the surrounding community [18]. Macroscopic morphological analysis of stomach contents is a widely used technique for prey identification. Despite limitations associated with the fast digestion of some prey items that could be just representative of short-term feeding [20] as it can provide information on intra- and interspecific relationships. Across the SO, where fisheries regularly occur, several dietary studies have been conducted using macroscopic and molecular prey identification [8,21], with recent studies using a combination of both providing new information that have improved the understanding of its trophic ecology [22]. This type of approach, complemented with other methods such as fatty acid analyses (lipids as biomarkers) has proven to be a powerful tool to infer trophic interactions and obtain longer-term information on energy flow through the ecosystem [22–25].

Feeding patterns described from TOA stomachs using both macroscopical prey composition and fatty acid profiles [23,24] have indicated that this species is an opportunistic predator, whose diet depends on the availability of prey in a given hábitat [16,26]. Based on this, current scientific consensus supports the idea of TOA as a generalist predator [19], whose diet composition varies ontogenetically, due to changes in vertical distribution throughout its life history [8,27].

Understanding the feeding ecology of Antarctic toothfish on the Antarctic Peninsula is crucial to assess its ecological role, which is key for ecosystem-based fishery management to avoid adverse indirect effects. A research program conducted by Ukraine in Subarea 48.1 allowed us to analyze stomachs collected over two consecutive summer seasons (2020 and 2021), and thus we did the first comprehensive study of feeding ecology through stomach content and fatty acid analyses. Here, we identified the prey composition of TOA in the AP using macroscopic guides and DNA for digested prey, tested the effect of size-class length, fishing season, and sex on diet variability, and characterized nutritional prey contribution from fatty acid profiles. We hypothesized that the diet composition changes with size-class length, showing an ontogenetic change in feeding habits.

**Materials and Methods**

Study area and sample collection

The stomach contents of 159 TOA individuals were analyzed from catches made by the Ukrainian commercial vessel Calipso in Subarea 48.1 (northern tip of the Antarctic Peninsula, Fig 1). Fishing operations were carried out in February 2020 (n = 89 stomachs) and February 2021 (n = 70 stomachs) using spanish bottom longlines at depths between 924-1560 meters in 2020 and between 1075-1371 meters in 2021 (S1 Table). Squid pieces were used as bait, not considered in subsequent stomach content analysis. Each individual TOA was weighted (gr), sexed and total length (cm) recorded onboard. Extracted stomachs were stored frozen at -20°C and sent for analysis to the Bioresources Laboratory of the Chilean Antarctic Institute (INACH) in Punta Arenas, Chile.

**Fig 1**. Locations of the fishing hauls carried out in the northern tip of the Antarctic Peninsula (Subarea 48.1). Boxes show CCAMLR research blocks 48.1_1 and 48.1_2, explored by the Ukrainian research program during 2020 and 2021.

Stomach content analysis

Whole stomachs were thawed, and content was weighed (mST) to the nearest 0.01 g using an electronic scale. Examination of prey included records of digestion status, taxonomic identification, wet weight (gr), length (cm), and fatty acid composition.

Digestion status was noted using a five-point scale: not digested, slightly digested, moderately digested, advanced digested, and heavily digested. Identification was to the lowest taxonomic level through macroscopic morphological analysis (mid) using identification keys [4,28]. Moderately to advanced digested prey were identified using genetic procedures (gid). Tissue samples from dorsal muscle were collected (∼10 mg) and stored in ethanol (90%) at -20°C. DNA extraction was performed with 0,150 g of tissue using DNeasy® PowerSoil® Pro (Cat.:47016) following the manufacturer’s protocol. Each sample was quantified using NanoQuant microplate Infinite M200pro. To amplify the mitochondrial 16s gene, primers were synthesized and purified by HPLC at Macrogen (Korea), using the sequence described by Berry et al. (2015) (forward: 5'-CGAGAAGACCCTRTGGAGCT-3' and reverse: 5'-GGATWGCGCTGTTATCCCT-3'). PCR was performed using Invitrogen^TM^ Platinum^TM^ SuperFi^TM^ II DNA Polymerase and the following thermocycling cycle: initial denaturation at 98°C for 5 min, 35 cycles of initial denaturation at 98°C for 10 s, annealing at 56°C for 5 s, extension at 72°C for 45 s, and a final extension at 72°C for 5 min. Each PCR product was run on a TBE 1,8 % agarose gel and purified using the UltraClean® 15 DNA Purification Kit (Cat.:12100-300), following the manufacturer’s protocol. The purified PCR products were quantified, and DNA sequencing was conducted in Macrogen using forward and reverse primers. The sequences obtained were joined and analyzed using the CLC Main Workbench (version 8.0). Taxonomic identification was performed using the NCBI database using a degree of similarity between the obtained sequences and reference sequences higher than 99%.

For fatty acid analysis, ten grams of prey muscle tissue was stored in 15 ml falcon tubes, frozen at -20° C and lyophilized. Samples were analyzed at the Nutrition Laboratory of the Department of Agricultural and Aquaculture Sciences of the Universidad de Magallanes (Punta Arenas, Chile). Qualitative and quantitative determination of fatty acids was carried out by transforming them into methyl esters (FAMES) and subjected to gas chromatographic analysis. Total lipids from the sample were estimated following the methodology proposed by [28] and then the fatty acids present in the sample were analyzed according to the methodology described by [29].

Extracted lipids were deposited in a 50 ml capacity balloon, to which 4 ml of methanolic solution of 0.5N sodium hydroxide were immediately added. The oxygen was then displaced from the balloon with a stream of nitrogen, refluxed in a batch with boiling water for 10 min, and 5 ml of 14% Boron Trifluoride (BF3-CH3OH, Sigma-Aldrich) was added to recirculation for 30 min. Then 2 ml of hexane was added, and almost immediately 20 ml of saturated sodium chloride (NaCl) was added and shaken vigorously for 15 sec. Saturated NaCl was added again up to the neck of the balloon, allowed to cool to room temperature, and finally, with a Pasteur pipette, the upper layer containing the methyl esters was extracted to be transferred to a vial for chromatography.

For gas chromatography analysis, an Agilent 7890B gas chromatograph equipped with an autosampler and FID detector was used. In addition, a HP-88 high polarity column (60 m x 0.20 µm x 0.25 mm) was used. The carrier gas (H2) flow rate was 1 ml/min and the flow split injection system with a 50:1 vent ratio was used. The injector temperature was 250 °C and the detector temperature was 280°C. The oven temperature program was 120°C for 5 min, with a ramp of 3°C/min up to 220°C (5 min). The injection volume was 1 µl and a blank was performed for every other analysis. All transesterification was performed in duplicate. Abbreviated notations of form A:B (n-x) were used, where A represents the number of carbon atoms, B the number of double bonds, and x gives the position of the first double bond counting from the terminal methyl group. The concentration of each fatty acid was expressed as the relative percentage of the total fatty acid content (% FAs) ± standard deviation.

Indicators of diet composition

The diet was analyzed only in samples with stomach contents, excluding empty stomachs. Diet composition and the importance of each prey item were calculated according to the percentage of frequency of occurrence (F%), weight (M%) and number (N%), respectively expressed as:

$F\%=\frac{Ai}{A} \cdot100 M\%= \frac{Mi}{Mt}\cdot100 N\%= \frac{Ni}{Nt}\cdot100$

Ai is the number of individuals that consumed prey i, and A is the total number of stomachs examined. Mi corresponds to the total wet weight of the prey i, and Mt is the total wet weight of the prey. Ni is the total number of prey i, and Nt is the total number of prey.

From these indicators, the importance of each type of prey within the diet was calculated using the index of relative importance (IRI) [30], expressed as:

$$IRI=\left( M\%+N\% \right)\cdot F\%$$

For a better interpretation of the relative contribution of each dam, we calculated the relative importance index as a percentage (IRI%) [31], expressed as:

$$IRI\%= \frac{{IRI}_{t}}{\sum_{i}^{n} IIR}\cdot100$$

In order to analyze diet among predator length, the IRI% was calculated into three size-class groups, G1: <100 cm, G2: 100-140 cm and G3: 140-180 cm.

Feeding intensity was evaluated according to the repletion index (RI) [32], expressed as:

$RI=\frac{mST}{mDM} \cdot100$

Where mST is the weight of the stomach contents, and mDM is the total weight of the individual.

Statistical analysis

A multivariate generalized linear model (MGLM) was used to test variability in the prey specific numeric abundance (N) according to sex, fishing season, and size-class. MGLM provides a multivariate test for the former factors and a univariate test for each prey item. The lowest Akaike’s information criterion (AIC) was selected to identify the model that best explained the amount of variation in N. MGLM was run with Poisson distribution and 999 Monte-Carlo permutations using mvabund v.4.2.1 R package [33]. To graphically visualize variability among factors, a non-metric multidimensional scaling ordination (nMDS) plot based on Bray-Curtis distances was run using vegan v.2.6-4 R package [34].

Differences in the repletion index were assessed between sex, fishing season, and size-class, using sqrt function in a one-way analysis of variance (ANOVA).

A permutational analysis of variance (PERMANOVA) test, based on Bray–Curtis distance matrix was used to assess for differences among FAs composition of prey composition. A similarity percentage analysis (SIMPER) was also used to identify the fatty acids contributing most to differences between prey items, and a nMDS plot was used to graphically illustrate observed patterns.

All statistical analyses were performed using Rstudio 2022.12.0+353. Map was drawn using ArcGIS 10.8.

**Results**

TOA individuals sampled in 2020 ranged from 64 to 174 cm TL (mean 125.1 ± 26.6 SD), with 70.5% females and 29.5% males. In 2021 sizes ranged from 66 to 176 cm TL (140.4 ± 24.8 cm), with 58.6% females and 41.4% males (Fig 2). From the 89 stomachs sampled in 2020, 75 (84.3%) had contents, with a total of 186 prey items (2.1 ± 2.1 items per stomach). Prey length ranged from 4.5 to 97 cm, exhibiting a significant positive predator-prey length relationship (ρ= 0.29, p < 0.01) (Fig 3). From the 70 stomachs collected in 2021, 58 (82.8%) had contents, with a total of 140 prey items (2.0 ± 2.0 prey items per stomach). Prey length ranged from 5 to 67 cm, with no significant predator-prey length relationship (ρ= 0.06, p = 0.69) (Fig 3).

In 2020, 4.9% of prey items were not digested, 14.2% slightly digested, 22.9% moderately digested, 39.8% advanced digested and 18.0% highly digested. In 2021, 5.5% of prey were not digested, 21.1% slightly digested, 27.5% moderately digested, 33.0% advanced digested, and 12.8% highly digested (S1 Fig).

**Fig 2**. Size structure of *Dissostichus mawsoni* individuals caught in the northern tip of the Antarctic Peninsula (Subarea 48.1) during the fishing seasons 2020 and 2021.

**Fig 3**. Size structure of prey items and its relationship with *Dissostichus mawsoni* individuals caught in the northern tip of the Antarctic Peninsula during fishing season 2020 (A-B) and 2021 (C-D).

**Diet composition**

Fifteen taxonomic groups were recorded from stomach contents. A total of 200 prey items were identified (168 mid, 32 gid) including fishes (*Anotopterus pharao,* *Chionobathyscus dewitti*, *Macrourus whitsoni*, *Muranolepis orangensis*, *Chionodraco rastrospinosus*, *Neopagetopsis ionah, Notothenia coriiceps*, *Trematomus eulepidotus, Lepidonotothen squamifrons*), crustaceans (*Eurythenes gryllus*, *Nematocarcinus lanceopes*), anthozoa, cephalopods, skates Rajidae and birds Spheniscidae (Table 1).

The TOA diet was dominated by fishes (91.0% in 2020, 86.3% in 2021) and secondarily by cephalopods (7.8% in 2020, 13.1% in 2021). Unidentified fish in advanced digestion accounted for 48.8% and 60.3% IRI for each fishing season, respectively. Among identified fishes, Macrouridae was the most important item (32.4% in 2020, 25.1% in 2021), followed by Anotopteridae (7.4%), Channichthyidae (3.0% in 2020, 0.4% in 2021) and Nototheniidae (0.1% in 2020, 0.03% in 2021). Crustacea, Rajidae, and penguin remain reached up 0.15%, 0.06% 0.03% IRI, respectively (Table 1).

The MGLM showed that the prey specific numeric abundance was best explained by the additive (non-interaction) effect, where size-class and fishing season showed significant effects (Table 2). Sex was discarded during the AIC selection process as it had no effect (S2 Table). As seen in the nMDS plot, diet variability was higher in G2 and G3 size groups than for G1 (Fig 4). Among identified prey, a significant effect of size-class was recorded on cephalopod consumption (Table 2) that was slightly present in groups G2 and G3 in 2020, being more relevant in 2021, especially G1 exceeding 55.0% IRI (Fig 5). On the other hand, a significant effect of the fishing season was found on Anotopteridae consumption (Table 2), which dominated G1 in 2020 (67.65% IRI) and was absent in 2021 (Fig 5). Although no significant effect of size group on other species was found, it can be seen that Macrouridae was less present in group G1 (29.99% IIR in 2020), but dominated the diet of groups G2 and G3 with 74.44% to 66.86% IRI, respectively, and Channichthyidae were particularly important to G1 in 2021 (28.87% IRI) (Fig 5)

Feeding intensity was low (Repletion Index = 1.23% ± 1.42 SD) with no significant differences between fishing season (ANOVA, F(1, 154) = 0.0004 p = 0.994), sex (ANOVA, F(1, 154) = 0.057, p= 0.812) and size classes (ANOVA, F(2, 153) = 0.102 p = 0.902).

**Table 1**. Diet composition of *Dissostichus mawsoni* in the northern tip of the Antarctic Peninsula (Subarea 48.1) during seasons 2020 and 2021, according to percentage frequency of occurrence (F%), mass percentage (M%), number percentage (N%) and the index of relative importance (IRI%). Parenthesis shows the number of prey items identified by macroscopic identification (mid) and genetic identification (gid).

|  | **2020** | | | | **2021** | | | |
| --- | --- | --- | --- | --- | --- | --- | --- | --- |
| **Prey** | **F%** | **N%** | **W%** | **IRI%** | **F%** | **N%** | **W%** | **IRI%** |
| Anthozoa *(mid=1)* | - | - | - | - | 1.72 | 0.71 | 0.01 | 0.02 |
| **Mollusca** | 26.6 | 12.90 | 4.45 | 7.88 | 27.59 | 20.00 | 13.08 | 13.17 |
| Cephalopoda *(mid=52)* | 26.67 | 12.90 | 4.45 | 7.88 | 27.59 | 20.00 | 13.08 | 13.17 |
| **Crustacea** | 5.33 | 3.23 | 0.11 | 0.15 | 6.90 | 4.29 | 0.16 | 0.15 |
| *Eurythenes gryllus (mid=3)* | - | - | - | - | 1.72 | 2.14 | 0.10 | 0.06 |
| *Nematocarcinus lanceopes (mid=6)* | 2.67 | 2.15 | 0.09 | 0.10 | 3.45 | 1.43 | 0.04 | 0.07 |
| Unidentified crustacea | 2.67 | 1.08 | 0.02 | 0.05 | 1.72 | 0.71 | 0.02 | 0.02 |
| **Pisces** | 90.67 | 83.33 | 95.16 | 91.95 | 93.10 | 72.14 | 84.54 | 86.33 |
| Anotopteridae | 26.67 | 15.59 | 4.91 | 7.39 | - | - | - | - |
| *Anotopterus pharao (mid=26, gid=03)* | 2.67 | 1.61 | 1.09 | 0.12 | - | - | - | - |
| Unidentified Anotopteridae | 24.00 | 13.98 | 3.81 | 7.27 | - | - | - | - |
| Channichthyidae *(mid=17, gid=5)* | 13.33 | 6.45 | 13.67 | 3.06 | 8.62 | 3.57 | 5.60 | 0.41 |
| *Chionobathyscus dewitti (gid=2)* | - | - | - | - | 3.45 | 1.43 | 1.88 | 0.16 |
| *Chionodraco rastrospinosus (gid=1)* | 1.33 | 0.54 | 1.65 | 0.05 | - | - | - | - |
| *Neopagetopsis ionah (gid=2)* | 1.33 | 0.54 | 1.01 | 0.04 | 1.72 | 0.71 | 1.15 | 0.05 |
| Unidentified Channichthyidae | 10.67 | 5.38 | 11.02 | 2.98 | 3.45 | 1.43 | 2.56 | 0.20 |
| Macrouridae | 48.00 | 23.66 | 54.62 | 32.46 | 43.10 | 22.14 | 53.22 | 25.13 |
| *Macrourus whitsoni (mid=17, gid=19)* | 17.33 | 8.06 | 28.98 | 10.93 | 25.86 | 15.00 | 36.36 | 19.16 |
| Unidentified Macrouridae | 30.67 | 15.59 | 25.63 | 21.52 | 17.24 | 7.14 | 16.86 | 5.97 |
| Muraenolepididae | 1.33 | 0.54 | 0.54 | 0.02 | 5.17 | 2.14 | 3.34 | 0.41 |
| *Muranolepis orangensis (gid=4)* | 1.33 | 0.54 | 0.54 | 0.02 | 5.17 | 2.14 | 3.34 | 0.41 |
| Nototheniidae | 2.67 | 2.69 | 4.38 | 0.16 | 1.72 | 0.71 | 0.38 | 0.03 |
| *Lepidonotothen squamifron*s (mid=4) | 1.33 | 2.15 | 3.56 | 0.13 | - | - | - | - |
| *Nothotenia coriiceps (gid=1)* | 1.33 | 0.54 | 0.82 | 0.03 | - | - | - | - |
| *Trematomus eulepidotus (gid=1)* | - | - | - | - | 1.72 | 0.71 | 0.38 | 0.03 |
| Rajidae | 1.33 | 0.54 | 2.06 | 0.06 | - | - | - | - |
| Unidentified Rajidae | 1.33 | 0.54 | 2.06 | 0.06 | - | - | - | - |
| Unidentified pisces | 58.67 | 33.87 | 14.98 | 48.80 | 63.79 | 43.57 | 22.01 | 60.35 |
| **Bird** | - | - | - | - | 5.17 | 2.14 | 2.07 | 0.31 |
| Spheniscidae *(mid=3)* | - | - | - | - | 5.17 | 2.14 | 2.07 | 0.31 |
| **Other** | 1.33 | 0.54 | 0.28 | 0.02 | 1.72 | 0.71 | 0.13 | 0.02 |
| Unidentified | 1.33 | 0.54 | 0.28 | 0.02 | 1.72 | 0.71 | 0.13 | 0.02 |

**Fig 4.** Scatterplot of nonmetric multidimensional scaling (nMDS) using Bray-Curtis distance matrix for prey specific numeric abundance among size-classes (G1: <0-100 cm (n=16); G2: 100-140 cm (n=64); G3: >140-180 cm (n=43)).

**Fig 5**. Diet composition of *Dissostichus mawsoni* according to size classes G1: <100 cm (n=16); G2: 100-140 cm (n=64); G3: >140 cm (n=43), collected in the northern tip of the Antarctic Peninsula (Subarea 48.1) during fishing seasons 2020 and 2021.

**Table 2**. Results of MGLM testing effect of size-class and fishing season on the prey specific numeric abundance in the stomachs of the Antarctic toothfish in the northern tip of the Antarctic Peninsula during fishing seasons 2019/20 and 2021/22. Ceph= Cephalopods, Ano= Anotopteridae, Cha= Channichthyidae, Mac= Macrouridae, Raj= Rajidae, Not= Nototheniidae, Mur= Muranoloepididae, Sph= Spheniscidae, Cru = Crustacea.

|  | **Multivariate** | | | | **Univariate** | | | | | | | | |
| --- | --- | --- | --- | --- | --- | --- | --- | --- | --- | --- | --- | --- | --- |
| **Factors** | **Res.Df** | **Df.diff** | **Dev** | **p (>Dev)** | **Cep** | **Ano** | **Cha** | **Mac** | **Raj** | **Not** | **Mur** | **Sph** | **Cru** |
| (Intercept) | 132 |  |  |  |  |  |  |  |  |  |  |  |  |
| Size class | 130 | 2 | 59.31 | **0.01*** | **0.01*** | 0.94 | 0.88 | 0.55 | 0.94 | 0.17 | 0.94 | 0.94 | 0.88 |
| Fishing season | 129 | 1 | 48.35 | **0.00*** | 0.87 | **0.00*** | 0.66 | 0.87 | 0.86 | 0.60 | 0.69 | 0.39 | 0.87 |

**Fatty acid composition of prey items**

A total of 29 fatty acids were identified for Anotopteridae, Channichthyidae, Macrouridae, and Cephalopoda. At the species level, we identified profiles for *Chionobathyscus dewitti* and *Macrourus whitsoni* (Table 3). Overall, prey ítems were rich in PUFA acids (ranging from 39.8% for Macrouridae to 49.3% for cephalopods), with a high contribution of docosexanoic acid (C22:6 n-3) and eicosapentanoic (C20:5 n-3). Secondarily, MUFA acids (ranging from 24.5% for cephalopods to 35.2% for Macrouridae) with oleic acid (C18:1 n-9) being higher in fishes (10.8% for Anotopteridae and 16.7% for Macrouridae) than cephalopods (5.4%), and conversely the eicosanoic acid (C20:1 n-9) higher in cephalopods (9.1%) than fishes (2.2% and 4.0% for Anotopteridae and Macrouridae, respectively). Thirdly, SAFA acids (ranging from 22.7% for Macrouridae to 24.8% for cephalopods), dominated by palmític acid (C16:0) of similar values for Macrouridae (16.8%), Anotopteridae (18.1%) and cephalopods (19.0%). The nMDS analysis revealed two prey groups of fishes (Anotopteridae-Macrouridae-Channichthyidae) and Cephalopoda (Fig 6), statistically different (F(4, 20)= 4.58; p<0.001). According to SIMPER analysis, the fatty acids that contribute most to dissimilarities between groups were oleic C18:1 n9cis, eicosanoic C20:1 n9, docosexanoic C22:6n3 DHA, linoleic C18:2 n6cis, palmitoleic C16:1n7 and octatetradenoic C18:4n3 (S3 Table).

**Table 3.** Fatty acid (FA) composition (% of total FA, mean ± SE) of prey items in the *Dissostichus mawsoni* diet from individuals collected in the northern tip of the Antarctic Peninsula. (SAFA are saturated fatty acids, MUFA are monounsaturated fatty acids, and PUFA are polyunsaturated fatty acids).

|  | **Macrouridae** | | **M. whitsoni** | | **Anotopteridae** | **Channichthyidae** | | **Cephalopoda** | |
| --- | --- | --- | --- | --- | --- | --- | --- | --- | --- |
|  | n = 9 | | n = 5 | | n = 1 | n = 2 | | n = 11 | |
| **Fatty acid** | mean | sd | mean | sd | mean | mean | sd | mean | sd |
| C14:0 | 1.91 | 0.46 | 1.63 | 0.47 | 1.90 | 3.62 | 4.19 | 2.48 | 0.82 |
| C15:0 | 0.02 | 0.07 | 0.00 | 0.00 | 0.00 | 0.16 | 0.23 | 0.01 | 0.03 |
| C16:0 | 16.83 | 3.14 | 18.59 | 1.46 | 18.10 | 15.82 | 1.28 | 19.06 | 2.56 |
| C17:0 | 0.00 | 0.00 | 0.00 | 0.00 | 0.00 | 0.00 | 0.00 | 0.02 | 0.07 |
| C18:0 | 3.77 | 0.92 | 4.62 | 0.38 | 3.70 | 3.07 | 2.21 | 3.15 | 0.95 |
| C21:0 | 0.19 | 0.38 | 0.00 | 0.00 | 0.00 | 0.00 | 0.00 | 0.08 | 0.27 |
| ***Ʃ SAFA*** | ***22.73*** | ***3.22*** | ***24.84*** | ***1.71*** | ***23.70*** | ***22.68*** | ***0.93*** | ***24.80*** | ***2.15*** |
| C14:1 | 0.02 | 0.07 | 0.00 | 0.00 | 0.00 | 0.25 | 0.36 | 0.00 | 0.00 |
| C16:1n7 | 5.77 | 2.09 | 4.09 | 1.26 | 3.60 | 6.68 | 6.11 | 1.92 | 1.33 |
| C17:1 | 0.00 | 0.00 | 0.00 | 0.00 | 0.00 | 0.00 | 0.00 | 0.03 | 0.09 |
| C18:1n9 | 0.00 | 0.00 | 0.00 | 0.00 | 0.00 | 0.00 | 0.00 | 0.01 | 0.03 |
| C18:1n9cis | 16.70 | 4.35 | 13.87 | 4.03 | 12.80 | 14.03 | 8.61 | 5.44 | 2.61 |
| C18:1n7 | 5.68 | 0.93 | 4.50 | 1.02 | 8.90 | 7.16 | 0.20 | 4.51 | 0.63 |
| C20:1 n9 | 4.04 | 2.29 | 3.54 | 1.64 | 2.20 | 1.73 | 0.16 | 9.11 | 1.11 |
| C22:1n9 | 1.93 | 0.86 | 2.06 | 0.59 | 2.30 | 2.89 | 3.09 | 3.22 | 1.17 |
| C24:1n9 | 1.10 | 0.32 | 1.46 | 0.17 | 1.00 | 1.03 | 0.89 | 0.27 | 0.30 |
| ***Ʃ MUFA*** | ***35.23*** | ***8.45*** | ***29.52*** | ***6.28*** | ***30.70*** | ***33.76*** | ***10.73*** | ***24.50*** | ***5.09*** |
| C16:3 n4 | 0.06 | 0.12 | 0.00 | 0.00 | 0.00 | 0.11 | 0.15 | 0.01 | 0.03 |
| C18:2n6 | 0.00 | 0.00 | 0.00 | 0.00 | 0.00 | 0.17 | 0.25 | 0.00 | 0.00 |
| C18:2n6cis | 1.38 | 0.21 | 1.21 | 0.19 | 1.30 | 2.04 | 0.48 | 0.14 | 0.28 |
| C18:3n3 | 0.12 | 0.23 | 0.00 | 0.00 | 0.00 | 0.55 | 0.78 | 0.00 | 0.00 |
| C18:4n3 | 0.84 | 0.42 | 0.59 | 0.53 | 0.00 | 1.31 | 1.86 | 0.09 | 0.22 |
| C18:3n6 | 0.01 | 0.03 | 0.00 | 0.00 | 0.00 | 0.00 | 0.00 | 0.00 | 0.00 |
| C20:2n6 | 0.03 | 0.10 | 0.00 | 0.00 | 0.00 | 0.00 | 0.00 | 0.91 | 0.67 |
| C20:3n6 | 0.01 | 0.03 | 0.00 | 0.00 | 0.00 | 0.00 | 0.00 | 0.00 | 0.00 |
| C20:3n3 | 0.00 | 0.00 | 0.26 | 0.59 | 0.00 | 0.00 | 0.00 | 0.12 | 0.20 |
| C20:4n6 | 1.04 | 0.79 | 0.59 | 0.46 | 0.00 | 0.44 | 0.23 | 1.69 | 1.16 |
| C20:4n3 | 0.12 | 0.24 | 0.11 | 0.15 | 0.30 | 0.19 | 0.27 | 0.00 | 0.00 |
| C20:5n3 | 14.22 | 4.00 | 14.93 | 1.63 | 13.80 | 15.65 | 4.44 | 16.92 | 2.03 |
| C22:5n3 | 0.98 | 0.23 | 1.30 | 0.34 | 1.50 | 0.64 | 0.07 | 0.98 | 0.57 |
| C22:6n3 | 21.03 | 6.01 | 25.99 | 4.19 | 28.40 | 20.34 | 12.82 | 28.51 | 7.89 |
| ***Ʃ PUFA*** | ***39.84*** | ***7.88*** | ***44.97*** | ***5.24*** | ***45.30*** | ***41.45*** | ***14.27*** | ***49.36*** | ***5.29*** |
| ***Ʃ FAs*** | ***97.78*** | ***2.61*** | ***99.31*** | ***1.09*** | ***99.70*** | ***97.90*** | ***2.60*** | ***98.65*** | ***1.50*** |
| Ʃ ω3 | 37.31 | 8.73 | 43.18 | 5.79 | 44.00 | 38.69 | 14.42 | 46.61 | 5.84 |
| Ʃ ω6 | 2.48 | 1.01 | 1.80 | 0.60 | 1.30 | 2.66 | 0.00 | 2.74 | 0.88 |
| ***ω3/ω6*** | ***15.06*** | ***8.65*** | ***27.34*** | ***12.78*** | ***33.85*** | ***14.56*** | ***5.42*** | ***19.08*** | ***7.79*** |

**Fig 6**. Scatterplot of nonmetric multidimensional scaling (nMDS) using Bray Curtis distance matrix for fatty acid data (expressed as the percentage of total fatty acids, %FAs) from the four identified prey groups recorded in stomachs of *Dissostichus mawsoni*.

**Discussion**

The present study provides the first comprehensive description of the feeding behavior of the Antarctic toothfish by combining stomach content identification with fatty acid analysis, providing a wider understanding of the feeding ecology and role of TOA in the Antarctic Peninsula. Trophic dynamics is a prerequisite for ecosystem-based fisheries management and necessary to assess potential fishing impacts on target species, which is especially true in the Antarctic Peninsula, where direct fishing for notothenioids has been prohibited.

**Feeding behavior**

In the Antarctic Peninsula, TOA feeds mainly on fishes, a prey of high nutritional value and often the most available, being usually the most important prey item for top predators in Antarctica [26,35]. Macrouridae was the most important prey, similar to what has been described in previous studies from other regions, where *Macrourus whitsoni* and *Macrourus caml*, a sympatric species inhabiting the same depth range with TOA (900 and 1900 m depth), frequently appears in TOA diet and as bycatch in the fishery [5,8,17,24,26,36–38]. Considering that the TOA diet is dominated by locally abundant fish species [5,39] and according to the high contribution of Macrouridae in the TOA diet observed in this study, we can assume that this group is probably the most abundant fish in the AP. Although there are no direct biomass estimations for Macrouridae in the AP, previous evidence showed this group as the most widespread representative of bycatch in 2020 and 2021 [40].

Channichthyidae was also an important prey. We identified *Chionobathyscus dewitti*, a species reported as one of the main prey of TOA in the Lazarev Sea and the Ross Sea [5,36], and commonly found between 600 to 1600 m depth in the Antarctic Peninsula [41,42], and over 2000 m depth in the Weddell Sea [42,43]. From the fishery data, Channichthyidae is the second most abundant bycatch group in the Antarctic Peninsula [40].

Surprisingly, we observed a very low contribution of Nototheniidae, even in individuals <100 cm, where it can reach up to 35% of the diet in individuals from East Antarctica [8]. It is possible that for greater energy efficiency, individuals prey on a smaller number of larger and heavier Macrouridae individuals, rather than a larger number of smaller and lighter Nototheniidae [8]. Another explanation could be that the fish community in the AP is heavily dominated by Macrouridae, with reduced availability of Nototheniidae as a consequence of the population collapse in past decades [15]. Although, there was an important amount of highly digested unidentified fish prey (which reached up to 60% IRI) where Nototheniidae could be present. Having said that, the reduced presence of Notheniidae was also observed in the fishery with low bycatch estimates in the area [40].

Although relatively rare, benthic crustaceans such as *Nematocarcinus lanceopes* and *Eurythenes gryllus* were also found. Both species have been recorded at depths between 500 and 2031 m [44,45], whereas *E. gryllus* has been reported between 550 to 7800 m depth [46,47], overlapping with TOA depth range. Another less important item was penguin remains (found in two stomachs), suggesting that part of the diet may have come from carrion. Also, we found coral and rock fragments, which is indicative of benthic foraging habits [8,17,26].

The former prey composition is representative of benthopelagic fauna [4,42], confirming TOA as a demersal feeder whose feeding preferences would be restricted to the abundance of different prey [5,8,17]. Also, the presence of *Anotopterus vorax* has been previously related to migration to shallow waters [26]. All this information confirms TOA as a top predator capable of impacting and controlling the bentho-pelagic ecosystem in the Antarctic Peninsula.

A positive correlation was observed between the TOA size and prey size, with prey size increasing as predator size increases. This is common as predator-prey interactions often depend on body size, due to the morphological limitations of the predator in agreement with the morphological ontogenetic changes in the feeding apparatus [48,49]. In other diet studies, TOA subadults show a diet composed of a variety of smaller prey, such as smaller fishes and benthic crustaceans, while adult individuals preyed mostly on larger demersal fishes, such as Macrouridae [17]. One point of variability was the presence of Anotopteridae, which, although exaggerated in length, can generally be considered small prey, impacting this predator-prey relationship.

Differences in diet composition were found between size groups, with dietary variability being greater in adults (>100 cm) than in juveniles (<100 cm). This can be related to changes in buoyancy and depth range distribution [27]. It is known that juveniles of TOA have a negative buoyancy, constraining depth range to shallower waters and benthic habitats, while adults have neutral buoyancy, being capable to move across a wider depth range from shallow to deep waters [4,6,17,27]. This adaptation would allow TOA to avoid predators and generate changes in accessibility to different prey items. Some prey, such as Cephalopoda and Channichthyidae, have benthic-pelagic habits [42,50], thus being important for juveniles. On the other hand, prey such as Macrouridae are abundant in deep waters [37], becoming more important for TOA adults. Other studies have also found changes in diet with predator length [5,8,17,51], which could demonstrate that there are ontogenetic changes in the diet of Antarctic toothfish. However, because all fishes caught in this study took place deeper than 900 m depth, juveniles were not represented and the smallest individuals analyzed were 80 cm; hence, it is necessary to extend to smaller sizes, probably on shallow waters, to obtain more determinant results.

Other sources of variability in TOA diet have been attributed to different areas and depth strata [8,22], but interannual variability is rarely analyzed. Considering that the TOA diet can strongly reflect local fish assemblages [39], it is interesting the changes in prey composition between two consecutive fishing seasons observed in this study. In Antarctica, demersal fish assemblages can vary according to water temperature [52], however this was not assessed in this study. The ongoing oceanographic changes well-described along the Antarctic Peninsula [53], could also be impacting the deep-water fish assemblage; hence it is important to measure physical factors to assess if they are driving changes in the distribution of prey in the area.

**Fatty acids prey composition**

Overall, PUFA was twice as numerous as MUFA and SAFA in all analyzed prey, which is typical in high latitude ecosystems than those from tropical [54]. We also observed higher content of w3 than w6 (high w3/w6 ratio), characteristic of marine ecosystems [55].

Fish and cephalopod prey showed similar amounts of saturated fatty acids (SAFAs), mostly dominated by palmític acid (C16:0), one of the most common FAs in marine organisms, key to providing metabolic energy during growth and spawning [56]. Among MUFAs there were higher values in fishes rather than cephalopods, especially oleic acid (C18:1), a fatty acid commonly found in prey that lives at great depths [57,58]. Both fishes and cephalopods were prey rich in PUFAs eicosapentaenoic (C20:5) and docosahexaenoic (C22:6), often considered essentials acids as most aquatic animals are not capable of synthesizing and are essential nutrient precursor, indicative of optimal animal health [56,59]. According to results, TOA feeds on prey with a high proportion of MUFAs and PUFAs, where both fishes and cephalopods can supply similar amounts of energy, so there could be no need of a specialist behavior, and potentially explaining the generalist strategy commonly associated with TOA feeding. Other predators tend to have a selective behavior as the differential energetic contribution among different prey [60]. Since we were not able to analyze predator tissues we could not compare the fatty acid signatures among the predator and their prey; this would have provided us with important information of quantitative trophic predator–prey relationship [61]. However, the information provided here still constitutes a key information to understanding energy flow and carbon transfer pathways in relation to the role of TOA in the benthic-demersal ecosystem of the Antarctic Peninsula.

**Management consideration**

Antarctic toothfish has been consistently managed by the CCAMLR as a key component in the food web dynamics of the Antarctic ecosystem [5,23]; however, issues including the population status, and ecosystem impacts of the fisheries are still to be well understood [62]. Moreover, climate change projections suggest that warming will negatively affect toothfish and other benthopelagic dwelling species [63]. For this reason, a complete understanding of feeding dynamics and trophic connections is relevant for ecosystem-based stock management in order to develop fishing activities while maintaining ecosystem structure and functioning. One risk in the toothfish fishery is the predation release, where fishing-induced changes in predator-prey relationship may generate the unbalanced proliferation of one species, destabilizing the structure and function of the food-web [22]. This process could be of particular concern in environments already modified by overexploitation, such as the Antarctic Peninsula, where it is unknown how TOA and the surrounding community will respond to fishing pressure, also considering the impacts of climate change in the area.

**Author Contributions**

**Conceptualization:** Karina Pérez-Pezoa, César A. Cárdenas, Marcelo González-Aravena, Francisco Santa Cruz

**Data curation:** Karina Pérez-Pezoa, Francisco Santa Cruz

**Formal analysis:** Karina Pérez-Pezoa, Francisco Santa Cruz

**Funding acquisition:** César A. Cárdenas, Marcelo González-Aravena

**Supervision:** César A. Cárdenas, Francisco Santa Cruz

**Visualization:** Karina Pérez-Pezoa, Francisco Santa Cruz

**Writing – original draft:** Karina Pérez-Pezoa, César A. Cárdenas, Marcelo González-Aravena, Pablo Gallardo, Alí Rivero, Vicente Arriagada, Kostiantyn Demianenko, Pavlo Zabroda, Francisco Santa Cruz

**Writing – review & editing:** César A. Cárdenas, Francisco Santa Cruz

**Acknowledgments**

The authors thank the crew and scientific observer of the Ukrainian vessel Calipso.

**Funding**

Funding for this study was provided by the Marine Protected Areas Program (Number 24 03 052) of the Instituto Antártico Chileno. CAC is also funded by ANID-Millennium Science Initiative Program—ICN2021_002.

**References**

1.	Eastman J. Antarctic fish biology: Evolution in a unique environment. Academic Press; 1993.

2.	Convey P, Peck LS. Antarctic environmental change and biological responses. Sci Adv. 2019;5. doi:10.1126/sciadv.aaz0888

3.	Barrera-Oro E. The role of fish in the Antarctic marine food web: differences between inshore and offshore waters in the southern Scotia Arc and west Antarctic Peninsula. Antarct Sci. 2002;14: 293–309. doi:10.1017/S0954102002000111

4.	Dewitt HH, Heemstra PC GO. Dewitt HH, Heemstra PC, Gon O. In: Gon O HP, editor. Fishes of the Southern Ocean. Smith Institute of Ichthyology; 1990.

5.	Fenaughty J, Stevens D HS. Diet of the Antarctic toothfish (Dissostichus mawsoni) from the Ross Sea, Antarctica (subarea 88.1). CCAMLR Sci. 2003;10: 113–123.

6.	Yates P, Ziegler P, Welsford D, Wotherspoon S, Burch P, Maschette D. Distribution of Antarctic toothfish Dissostichus mawsoni along East Antarctica: Environmental drivers and management implications. Fish Res. 2019;219: 105338. doi:10.1016/j.fishres.2019.105338

7.	Eastman JT, DeVries AL. Aspects of body size and gonadal histology in the Antarctic toothfish, Dissostichus mawsoni , from McMurdo Sound, Antarctica. Polar Biol. 2000;23: 189–195. doi:10.1007/s003000050026

8.	Seong GC, Choi SG, Chung S, An DH, Kim HW, Baeck GW. Morphological dietary composition of Antarctic toothfish (Dissostichus mawsoni) along the East Antarctic continental slope. Polar Biol. 2021;44: 499–508. doi:10.1007/s00300-021-02820-9

9.	Hanchet S, Dunn A, Parker S, Horn P, Stevens D, Mormede S. The Antarctic toothfish (Dissostichus mawsoni): biology, ecology, and life history in the Ross Sea region. Hydrobiologia. 2015;761: 397–414. doi:10.1007/s10750-015-2435-6

10.	Yukhov V. The range of Dissostichus mawsoni Norman and some features of its biology. J Ichthyol. 1971;11: 14–25.

11.	Calhaem I, Christoffel DA. Some observations of the feeding habits of a Weddell seal, and measurements of its prey, Dissostichus mawsoni , at McMurdo Sound, Antarctica. New Zeal J Mar Freshw Res. 1969;3: 181–190. doi:10.1080/00288330.1969.9515287

12.	Drazen JC, Sutton TT. Dining in the Deep: The Feeding Ecology of Deep-Sea Fishes. Ann Rev Mar Sci. 2017;9: 337–366. doi:10.1146/annurev-marine-010816-060543

13.	Hanchet S, Sainsbury K, Butterworth D, Darby C, Bizikov V, Rune Godø O, et al. CCAMLR’s precautionary approach to management focusing on Ross Sea toothfish fishery. Antarct Sci. 2015;27: 333–340. doi:10.1017/S095410201400087X

14.	CCAMLR. Statistical Bulletin, Vol. 34. 2022. Available: www.ccamlr.org

15.	Marschoff ER, Barrera-Oro ER, Alescio NS, Ainley DG. Slow recovery of previously depleted demersal fish at the South Shetland Islands, 1983–2010. Fish Res. 2012;125–126: 206–213. doi:10.1016/j.fishres.2012.02.017

16.	Fenaughty JM, Stevens DW, Hanchet SM. Diet of the Antarctic toothfish (Dissostichus mawsoni) from the Ross Sea, Antarctica (Subarea 88.1). CCAMLR Sci. 2003;10: 113–123.

17.	Stevens DW, Dunn MR, Pinkerton MH, Forman JS. Diet of Antarctic toothfish ( Dissostichus mawsoni ) from the continental slope and oceanic features of the Ross Sea region, Antarctica. Antarct Sci. 2014;26: 502–512. doi:10.1017/S095410201300093X

18.	Sallaberry-Pincheira P, Galvez P, Molina-Burgos BE, Fernandoy F, Melendez R, Klarian SA. Diet and food consumption of the Patagonian toothfish (Dissostichus eleginoides) in South Pacific Antarctic waters. Polar Biol. 2018;41: 2379–2385. doi:10.1007/s00300-018-2360-z

19.	Soeffker M, Riley A, Belchier M, Teschke K, Pehlke H, Graham J, Somhlaba S, Namba T DC. Towards the Development of a Stock Hypothesis for Antarctic Toothfish (Dissostichus mawsoni) in area 48. 2018. Report No.: WG-SAM-18/33.

20.	da Silveira EL, Semmar N, Cartes JE, Tuset VM, Lombarte A, Ballester ELC, et al. Methods for Trophic Ecology Assessment in Fishes: A Critical Review of Stomach Analyses. Rev Fish Sci Aquac. 2020;28: 71–106. doi:10.1080/23308249.2019.1678013

21.	Yoon T-H, Kang H-E, Lee SR, Lee J-B, Baeck GW, Park H, et al. Metabarcoding analysis of the stomach contents of the Antarctic Toothfish ( Dissostichus mawsoni ) collected in the Antarctic Ocean. PeerJ. 2017;5: e3977. doi:10.7717/peerj.3977

22.	Queirós JP, Stevens DW, Pinkerton MH, Rosa R, Duarte B, Baeta A, et al. Feeding and trophic ecology of Antarctic toothfish Dissostichus mawsoni in the Amundsen and Dumont D’Urville Seas (Antarctica). Hydrobiologia. 2022;849: 2317–2333. doi:10.1007/s10750-022-04871-3

23.	Jo H, Yeon I LC. Fatty acid and stable isotope analyses to infer diet of Antarctic toothfish caught in the southern Ross Sea. CCAMLR Sci. 2013;20: 1–19.

24.	Park H, Yeon I, Lee Y HE. Diet study of Antarctic toothfish caught in the east Antarctic based on stomach content, fatty acid and stable isotope analyses. CCAMLR Sci. 2015;22: 29–44.

25.	Moreira E, Novillo M, Mintenbeck K, Alurralde G, Barrera-Oro E, De Troch M. New insights into the autecology of the two sympatric fish species Notothenia coriiceps and N. rossii from western Antarctic Peninsula: A trophic biomarkers approach. Polar Biol. 2021;44: 1591–1603. doi:10.1007/s00300-021-02903-7

26.	Roberts J, Xavier JC, Agnew DJ. The diet of toothfish species Dissostichus eleginoides and Dissostichus mawsoni with overlapping distributions. J Fish Biol. 2011;79: 138–154. doi:10.1111/j.1095-8649.2011.03005.x

27.	Near TJ, Russo SE, Jones CD, DeVries AL. Ontogenetic shift in buoyancy and habitat in the Antarctic toothfish, Dissostichus mawsoni (Perciformes: Nototheniidae). Polar Biol. 2003;26: 124–128. doi:10.1007/s00300-002-0459-7

28.	Bligh EG, Dyer WJ. A RAPID METHOD OF TOTAL LIPID EXTRACTION AND PURIFICATION. Can J Biochem Physiol. 1959;37: 911–917. doi:10.1139/o59-099

29.	Metcalfe LD, Schmitz AA, Pelka JR. Rapid Preparation of Fatty Acid Esters from Lipids for Gas Chromatographic Analysis. Anal Chem. 1966;38: 514–515. doi:10.1021/ac60235a044

30.	Pinkas L, Oliphant, Iverson M IL. Food habits of albacore, bluefin tuna, and bonito in California waters. 1971.

31.	Cortés E. A critical review of methods of studying fish feeding based on analysis of stomach contents: application to elasmobranch fishes. Can J Fish Aquat Sci. 1997;54: 726–738. doi:10.1139/f96-316

32.	Berg J. Discussion of methods of investigating the food of fishes, with reference to a preliminary study of the prey of Gobiusculus flavescens (Gobiidae). Mar Biol. 1979;50: 263–273. doi:10.1007/BF00394208

33.	Wang Y, Naumann U, Wright ST, Warton DI. mvabund - an R package for model-based analysis of multivariate abundance data. Methods Ecol Evol. 2012;3: 471–474. doi:10.1111/j.2041-210X.2012.00190.x

34.	Oksanen, J, Kindt, R, Legendre, P, O’hara, B, Stevens, MHH, Oksanen, MJ, Suggests M. The vegan package: community ecology package. 2007.

35.	Kock KH. Studies in polar research: Antarctic fish and fisheries. Cambridge University Press; 1994.

36.	Petrov AF, Tatarnikov VA. Results of investigation of the diet of antarctic toothfish Dissostichus mawsoni (Nototheniidae) in the Lazarev Sea. J Ichthyol. 2011;51: 131–135. doi:10.1134/S0032945210051017

37.	Pinkerton M, McMillan P FJ. Distribution, morphology and ecology of Macrourus whitsoni and M. caml (Gadiformes, Macrouridae) in the Ross Sea region. CCAMLR Sci. 2013;20: 37–61.

38.	Pinkerton MH, Bradford-Grieve JM. Characterizing foodweb structure to identify potential ecosystem effects of fishing in the Ross Sea, Antarctica. ICES J Mar Sci. 2014;71: 1542–1553. doi:10.1093/icesjms/fst230

39.	Lee SR, Choi S-G, Chung S, Kim DN, Kang C-K, Kim H-W. Geographical differences in the diet of Dissostichus mawsoni revealed by metabarcoding. Front Mar Sci. 2022;9. doi:10.3389/fmars.2022.888167

40.	WG-SAM-17. Report on the toothfish survey in the Subarea 48.1 by the Ukrainian vessel Calipso in 2021. 2021.

41.	Arana PM, Vega R. Exploratory fishing for Dissostichus spp. in the Antarctic region (Subareas 48.1, 48.2 and 88.3). CCAMLR Sci. 1999;6: 1–17.

42.	Kock K-H. Antarctic icefishes (Channichthyidae): a unique family of fishes. A review, Part I. Polar Biol. 2005;28: 862–895. doi:10.1007/s00300-005-0019-z

43.	Hureau J-C. The significance of fish in the marine Antarctic ecosystems. Polar Biol. 1994;14. doi:10.1007/BF00238445

44.	Arntz WE, Gorny M. Shrimp (Decapoda, Natantia) occurrence and distribution in the eastern Weddell Sea, Antarctica. Polar Biol. 1991;11. doi:10.1007/BF00240205

45.	Gutt J, Ekau W GM. New results on the fish and shrimp fauna of the Weddell Sea and Lazarev Sea (Antarctic). Proc NIPR Symp Polar Biol. 1994;7: 91–102.

46.	Bowman TE, Manning RB. Two Arctic Bathyal Crustaceans: the Shrimp Bythocaris Cryonesus New Species, and the Amphipod Eurythenes Gryllus, With in Situ Photographs From Ice Island T-3. Crustaceana. 1972;23: 187–201. doi:10.1163/156854072X00363

47.	Thurston MH, Petrillo M, Della Croce N. Population structure of the necrophagous amphipod Eurythenes gryllus (Amphipoda: Gammaridea) from the Atacama Trench (south-east Pacific Ocean). J Mar Biol Assoc United Kingdom. 2002;82: 205–211. doi:10.1017/S0025315402005374

48.	Paine RT. Size-Limited Predation: An Observational and Experimental Approach with the Mytilus-Pisaster Interaction. Ecology. 1976;57: 858–873. doi:10.2307/1941053

49.	Olson MH. Predator-prey interactions in size-structured fish communities: implications of prey growth. Oecologia. 1996;108: 757–763. doi:10.1007/BF00329052

50.	Queirós JP, Ramos JA, Cherel Y, Franzitta M, Duarte B, Rosa R, et al. Cephalopod fauna of the Pacific Southern Ocean using Antarctic toothfish (Dissostichus mawsoni) as biological samplers and fisheries bycatch specimens. Deep Sea Res Part I Oceanogr Res Pap. 2021;174: 103571. doi:10.1016/j.dsr.2021.103571

51.	Takahashi M, Iwami T. The summer diet of demersal fish at the South Shetland Islands. Antarct Sci. 1997;9: 407–413. doi:10.1017/S0954102097000527

52.	Clark MR, Dunn MR, McMillan PJ, Pinkerton MH, Stewart A, Hanchet SM. Latitudinal variation of demersal fish assemblages in the western Ross Sea. Antarct Sci. 2010;22: 782–792. doi:10.1017/S0954102010000441

53.	Wang X, Moffat C, Dinniman MS, Klinck JM, Sutherland DA, Aguiar‐González B. Variability and Dynamics of Along‐Shore Exchange on the West Antarctic Peninsula (WAP) Continental Shelf. J Geophys Res Ocean. 2022;127. doi:10.1029/2021JC017645

54.	Colombo SM, Wacker A, Parrish CC, Kainz MJ, Arts MT. A fundamental dichotomy in long-chain polyunsaturated fatty acid abundance between and within marine and terrestrial ecosystems. Environ Rev. 2017;25: 163–174. doi:10.1139/er-2016-0062

55.	Iverson SJ. Tracing aquatic food webs using fatty acids: from qualitative indicators to quantitative determination. Lipids in Aquatic Ecosystems. New York, NY: Springer New York; 2009. pp. 281–308. doi:10.1007/978-0-387-89366-2_12

56.	Tocher DR. Metabolism and Functions of Lipids and Fatty Acids in Teleost Fish. Rev Fish Sci. 2003;11: 107–184. doi:10.1080/713610925

57.	Stowasser G, McAllen R, Pierce GJ, Collins MA, Moffat CF, Priede IG, et al. Trophic position of deep-sea fish—Assessment through fatty acid and stable isotope analyses. Deep Sea Res Part I Oceanogr Res Pap. 2009;56: 812–826. doi:10.1016/j.dsr.2008.12.016

58.	Parzanini C, Parrish CC, Hamel J-F, Mercier A. Functional diversity and nutritional content in a deep-sea faunal assemblage through total lipid, lipid class, and fatty acid analyses. Loor JJ, editor. PLoS One. 2018;13: e0207395. doi:10.1371/journal.pone.0207395

59.	Parrish CC. Essential fatty acids in aquatic food webs. Lipids in Aquatic Ecosystems. New York, NY: Springer New York; 2009. pp. 309–326. doi:10.1007/978-0-387-89366-2_13

60.	Kohl KD, Coogan SCP, Raubenheimer D. Do wild carnivores forage for prey or for nutrients? BioEssays. 2015;37: 701–709. doi:10.1002/bies.201400171

61.	Zhang J, Ren C, Zhang H, Yin F, Zhang S, Wan R, et al. Review of Estimating Trophic Relationships by Quantitative Fatty Acid Signature Analysis. J Mar Sci Eng. 2020;8: 1030. doi:10.3390/jmse8121030

62.	Brooks CM, Ainley DG, Jacquet J, Chown SL, Pertierra LR, Francis E, et al. Protect global values of the Southern Ocean ecosystem. Science (80- ). 2022;378: 477–479. doi:10.1126/science.add9480

63.	Chown, S.L., Leihy, R.I., NaishChown, S.L., Leihy, R.I., Naish, T.R., Brooks, C.M., Convey, P., Henley, B.J., Mackintosh, A.N., Phillips, L.M., Kennicutt, M.C. II & Grant, S.M., T.R., Brooks, C.M., Convey, P., Henley, B.J., Mackintosh, A.N., Phillips, L.M SM. Antarctic Climate Change and the Environment: A Decadal Synopsis and Recommendations for Action. Cambridge; 2022. Available: www.scar.org
