# Supplementary material for: Trophodynamics of the Antarctic toothfish (Dissostichus mawsoni) in the Antarctic Peninsula: Ontogenetic changes in diet composition and prey fatty acid profiles
Source: PLoS One. 2023 Oct 5;18(10):e0287376. doi: 10.1371/journal.pone.0287376 (PMC10553334; doi:10.1371/journal.pone.0287376)
Supplement: Attachment — Submitted filename: Perez-Pezoa_etal_PONE_Response to Reviewers.docx [file pone.0287376.s009.docx]

Ref.:  MS PONE-D-23-08000

Title: Trophodynamics of the Antarctic toothfish (*Dissostichus mawsoni*) in the Antarctic Peninsula: Ontogenetic changes in diet composition and prey fatty acid profiles

Dear reviewers,

Thank you for your constructive review of our study that definitely contributed to improving the manuscript. Responses below describe changes based on all comments. In the review letter, we refer to the text indicating the lines of the clean version of the MS, rather than the mark-up version. We hope you find our changes satisfactory.

***Reply to Reviewers***

| Reviewer #1 | | |
| --- | --- | --- |
| I have checked the paper entitled "Trophodynamics of the Antarctic toothfish (Dissostichus mawsoni) in the Antarctic Peninsula: Ontogenetic changes in diet composition and prey fatty acid profiles" The paper was very good and well written but needed some corrections as below. | | |
| ***Page/line, or section*** | ***Comment*** | ***Reply*** |
| Methods | For molecular analyses, you reached the phylogenetic tree or only by BLAST, the sequences checked, and what species compared with? | Genetic taxonomic identification was using BLAST with a 99% degree of similarity between the obtained sequences and reference sequences in the NCBI database. Please see this between lines 157 to 159. |
| Figure 3 | In Fig. 3. R2 ratio is so weak; do you have any explanation for it? | This weak relationship is explained by the presence of Anotopteridae, a long but slender prey that is preyed by small TOA. We addressed this between lines 383 and 386. |
| Reviewer #2 | | |
| Recent study on "Trophodynamics of the Antarctic toothfish (Dissostichus mawsoni) in the Antarctic  Peninsula: Ontogenetic changes in diet composition and prey fatty acid profiles" base on Prey- Predator relationship in aquatic ecosystem is reliable which gives the information about the food chain also. | | |
| ***Page/line, or section*** | ***Comment*** | ***Reply*** |
| Results | The study of feeding material or prey based on molecular basis is give the trustable impact of the effective study But in graphical representation figure no.3 between in numbers and length is confusable regarding the depending and independing variable (length,Number) for choosing the coordinates (X and Y). So please check it and see the manuscript which is attach with minor correction in Graph. | Figure 3A and 3C are histograms representing the frequency of occurrence (counts) of the sampled individuals along a size range. There is no dependence between variables. |
| Reviewer #3 | | |
| I have evaluated the manuscript “PONE-D-23-08000 Trophodynamics of the Antarctic toothfish (Dissostichus mawsoni) in the Antarctic Peninsula: Ontogenetic changes in diet composition and prey fatty acid profiles. I am suggesting the authors and curious to know the some major observations. The manuscript is well written and results are well described. Data were well statistically analysed. Results are properly discussed in discussion section. The authors need to revise manuscript in following given points. I am very satisfied with work done by authors and recommending the manuscript after the completion of the revision. | | |
| ***Page/line, or section*** | ***Comment*** | ***Reply*** |
| Methods | Authors did two sampling in two successive years in the month of February. What was the reason for choosing the month February? Why other months were not chosen for sampling? Whether two samplings are enough to determine the feeding habits of different species? Explain it. | Surely more than two years is needed to fully understand the trophic ecology of this species in the Antarctic Peninsula. We would like to extend these analyses to other months, but this study was part of an "exploratory fishery" that Ukraine is conducting in CCAMLR waters, and February is the month with the best climatic conditions for fishing activity. |
| Methods | Authors dissected out the stomach of different samples and classified the prey items based on the extent of digestion in stomach. Digestion starts in stomach and only protein is digested in stomach. Then how can you have classified them in different extent of digestion? In this scenario, how did you take the sample of these preys? And if prey were gone digestion process, whether it represented the actual fatty composition? Authors have performed fatty acid composition and correlated with nutritional quality of prey. What was the reason behind it? How can authors correlates the fatty acid composition with prey’s nutritional quality. | We classified prey according to 5-point scale described between lines 135 to 138.  Only prey up to moderately digested status were used for FA analyses. Please see lines 138 to 139.  We assumed that fatty acid composition and especially the presence and relative contribution of some essential fatty acids such as EPA or DHA, are representative of the nutritional value of a prey. We supported that assumption following literature both in the Introduction and the properly discussed. Please see details in lines 87 to 92 (Introduction), and 424 to 436 (Discussion). |
| Methods | Line number 169. Expression of fatty acid composition is shown as % total fatty acid content? It should be checked and should be expressed per kg of total lipid. | We used the direct transesterification method to calculate fatty acids composition (Lepage and Roy 1986, and modified by Rodriguéz-Ruiz et al., 1998). We did not calculate the total lipid expressed in g/kg. This was described between lines 160 to 172. |
| Methods | Standard notations for units should be used. For instance, author used gr as notation for gram. It should be g | Done |
| Methods | Line no. 128. Cite the reference for DNA extraction method. As the kit used for DNA content analysis is based on someone's protocol/ principle | Done |
| Methods | Line no 135. Is It 1.8% agar or 18% agar. Check it. | 1.8% |
| Methods | Line number 161- H2, should be changed to H2. | Done |
| Conclusion | Authors need to write a profound conclusion. | Thanks. Agreed. We included a section with the relevant conclusions of our work. Please see lines 466 to 480. |
| Reviewer #4 | | |
| This study describes the feeding preferences of a generalist predator in the Antarctic Peninsula after a population collapse following overexploitation. Conclusions surrounding relative abundance of prey species are drawn from quantity of prey found in diet of TOA over two years of fishing. I find this study well written and interesting and would recommend it for publication after minor changes listed below. | | |
| ***Page/line, or section*** | ***Comment*** | ***Reply*** |
| Abstract | “Other rare prey found”, infers that the prey listed previously was also rare? I assume that ‘rare’ refers to rare diet choice as opposed to rare species. Please clarify. | We modified to clarify between lines 32 to 33. |
| Introduction | Line 54: change to ‘The TOA is by far the…’ | Thanks. Done. |
| Introduction | Line 79: be clearer with what you mean by ‘short-term feeding’, i.e., opportunistic feeding? That prey was therefore likely just ingested but not assimilated? | We meant that macroscopic morphological analysis has limitations as some prey are quickly digested and could be undetected, and therefore underestimated. Please see lines 78 to 80. |
| Introduction | Lines 87-92: But what is still lacking from these previous studies? Why do this study? | The main reason is the lack of trophic studies of this species in the Antarctic Peninsula, since most of the samples come from other waters where the fishery operates regularly. Please see lines xx to xx. |
| Methods | Fig.1: what do the coloured areas (red and yellow hatched lines) represent? | Its represent the two fishing blocks implemented by CCAMLR for exploratory fisheries. We detailed that in the caption of Figure 1. |
| Methods | Line 123: what does ‘(mid)’ refer to here? Is this an acronym? If so, please consider capitalising. Same applies to ‘(gid)’ in line 124. | Done. |
|  | Line 125: was it always possible to identify dorsal muscle from individual prey that have been highly digested? | No always. We only used muscle from moderately or advanced digested prey for genetic identification. Please see lines 141 to 142. |
|  | Line 128: any minimum DNA concentration used before sending? | 10 ng. Please see line 143. |
|  | Line 213: might be more informative if you report SD without the inclusion of the ~17% of fish with no stomach contents. | SD was reported without empty stomachs. |
|  | Line 215: where did these results (ρ= 0.29, p < 0.01) come from? I don’t see them in Figure 3. What does ρ refer to? | We miss that. Please see lines 207 to 208 where we detailed a Spearman’s correlation test used. |
|  | Line 218: Specify Fig. 3c,d for 2021 (and 3a,b for 2020 in line 215) | Done |
|  | Line 222/Methods section: was it the same person recording how digested prey items were? I.e. was there inter-observer variability in this subjective measurement? | Yes. Was the same person (the first author) who did this job alone, without inter-observations |
|  | Table 1: missing W% from figure legend, and missing M% from table – mixed up? Would be good to have a thicker line separating the 2 years to make reading the table easier. | Done |
|  | Table 2/Line 244: Although significant, which size class (i.e. G1,G2 or G3) and/or fishing season (2021 or 2021) had the most effect on prey specific numeric abundance? Post-hoc comparisons would be useful here. | We compared size classes by using a pairwise.comp of the anova.manyglm function (line 214). We identified that major differences are between groups G2 and G3 (line 267 to 268 and the Supplementary Table S3). Fishing season were not compared as this analysis required a minimum of three categories. |
|  | Line 244: sex does have an effect but was not included in the best model (as determined from AIC). It is also likely confounded with size class as well, especially in 2021. | Sex showed not significant effect, thus was discarded during the AIC selection process in order to obtain the most parsimonious model. Please see lines 268 to 271. |
|  | Table S2: define what ΔAIC is in the legend. | Done |
|  | Fig. 4: would be good to arrange your legend in order of the size classes to make it clearer. Also label which is G1, G2 and G3. | Done |
|  | Discussion: would be good to expand upon the specific impacts of climate change on notothenioid species since this is often referred to throughout the manuscript, but only touched upon in the discussion. | Agreed. We expanded that between lines 448 to 464. |
